# Taxonomic Assessment of Rumen Microbiota Using Total RNA and Targeted Amplicon Sequencing Approaches

**DOI:** 10.3389/fmicb.2016.00987

**Published:** 2016-06-22

**Authors:** Fuyong Li, Gemma Henderson, Xu Sun, Faith Cox, Peter H. Janssen, Le Luo Guan

**Affiliations:** ^1^Department of Agricultural, Food and Nutritional Science, University of AlbertaEdmonton, AB, Canada; ^2^AgResearch Ltd., Grasslands Research CentrePalmerston North, New Zealand

**Keywords:** total RNA sequencing, targeted amplicon sequencing, rumen microbiota, bacteria, archaea

## Abstract

Taxonomic characterization of active gastrointestinal microbiota is essential to detect shifts in microbial communities and functions under various conditions. This study aimed to identify and quantify potentially active rumen microbiota using total RNA sequencing and to compare the outcomes of this approach with the widely used targeted RNA/DNA amplicon sequencing technique. Total RNA isolated from rumen digesta samples from five beef steers was subjected to Illumina paired-end sequencing (RNA-seq), and bacterial and archaeal amplicons of partial 16S rRNA/rDNA were subjected to 454 pyrosequencing (RNA/DNA Amplicon-seq). Taxonomic assessments of the RNA-seq, RNA Amplicon-seq, and DNA Amplicon-seq datasets were performed using a pipeline developed in house. The detected major microbial phylotypes were common among the three datasets, with seven bacterial phyla, fifteen bacterial families, and five archaeal taxa commonly identified across all datasets. There were also unique microbial taxa detected in each dataset. *Elusimicrobia* and *Verrucomicrobia* phyla; *Desulfovibrionaceae, Elusimicrobiaceae*, and *Sphaerochaetaceae* families; and *Methanobrevibacter woesei* were only detected in the RNA-Seq and RNA Amplicon-seq datasets, whereas *Streptococcaceae* was only detected in the DNA Amplicon-seq dataset. In addition, the relative abundances of four bacterial phyla, eight bacterial families and one archaeal taxon were different among the three datasets. This is the first study to compare the outcomes of rumen microbiota profiling between RNA-seq and RNA/DNA Amplicon-seq datasets. Our results illustrate the differences between these methods in characterizing microbiota both qualitatively and quantitatively for the same sample, and so caution must be exercised when comparing data.

## Introduction

Microbiota play essential roles in many ecosystems, including the animal gastrointestinal tract, and have attracted much attention in the past decade due to the understanding of their functions in host productivity and health (Holmes et al., 2012; Million et al., 2013; Yeoman and White, 2014). Numerous studies have shown that changes in gastrointestinal microbiota at the taxonomic and/or functional levels are associated with host dysfunction and metabolic diseases (Marchesi et al., 2016; Ojeda et al., 2016), highlighting the importance of studying the interactions that exist between gastrointestinal microbiota and host animals. Therefore, an accurate assessment of the composition and diversity of rumen microbiota is essential to link microbiota changes to host performance under different conditions.

To date, culture-independent molecular-based taxonomic assessment of microbiota has primarily relied on the sequencing of PCR amplicons of targeted microbial genes at the DNA level (DNA Amplicon-seq). Although Amplicon-seq has been widely used, it can be biased due to primer selection (Hong et al., 2009) and/or amplification cycling conditions (Huber et al., 2009). It is also limited in discovering novel microbial phylotypes because the associated primers are designed based on known sequences (Urich et al., 2008; Ross et al., 2012). In addition, to study different groups of microbes within the same microbiota, a wide range of primers is needed (Kittelmann et al., 2013). Total DNA sequencing (metagenomics) has also been widely used to study microbiota without PCR amplification, and provides information on the presence and absence of phylotypes, but it mainly offers insight in microbial functions through studying microbiota-associated genes. Although a couple of studies have assessed microbial profiles based on 16S rDNA sequences generated in metagenomics datasets (Ellison et al., 2014; Logares et al., 2014), most metagenomic studies rely on parallel DNA Amplicon-seq to characterize microbial communities (Mason et al., 2014; Rooks et al., 2014) due to the low fraction of 16S rDNA reads present in metagenomics datasets (Logares et al., 2014). Meanwhile, DNA-based methods do not directly measure the activity of the microbiota because they cannot distinguish the presence of genes that stem from active cells, inactive but alive cells, dead cells, or lysed cells (Gaidos et al., 2011).

To overcome these limitations of DNA-based approaches, recent improvements in RNA sequencing have created a great opportunity to study potentially active microbiota. However, RNA sequencing has mainly been applied to elucidate the functions of microbiota through mRNA enrichment (de Menezes et al., 2012; Franzosa et al., 2014) and to study active phylotypes through 16S rRNA amplicon sequencing (RNA Amplicon-seq; Gaidos et al., 2011; Kang et al., 2013). Total RNA sequencing (RNA-seq) has been explored for taxonomic assessment of microbial communities in a number of environments, including soil (Urich et al., 2008; Tveit et al., 2014), hydrothermal vents (Lanzen et al., 2011), and the animal gut (Poulsen et al., 2013; Schwab et al., 2014). However, most of these studies did not compare outcomes between DNA- and RNA-based methods for the same samples and did not compare RNA-seq vs. Amplicon-seq (DNA/RNA), except Berry et al. (2012), who used DNA Amplicon-seq and RNA-seq to study shifts in murine gut microbiota in dextran sodium sulfate (DSS)-induced colitis, and Lanzen et al. (2011), who explored microbial communities at both the DNA and RNA levels in the hydrothermal vents. To date, it is not conclusive which method is the most reliable to assess animal gastrointestinal microbial communities because the different outcomes of these methods have not yet been comprehensively compared.

In this study, we compared bacterial and archaeal community profiles in rumen digesta samples using RNA-seq and RNA/DNA Amplicon-seq with standard protocols and a pipeline developed in house. The rumen microbial community is complex and includes bacteria, archaea, protozoa and fungi (Edwards et al., 2004). Although Poulsen et al. (2013) used RNA-seq to study rumen microbiota, they mainly focused on methanogens and did not analyze bacteria or compare RNA-seq with Amplicon-seq (DNA and RNA). In the current study, our aim was to gain a better understanding of the differences between the techniques using different genetic materials (DNA vs. RNA) and how they affect interpretation of microbiota-associated data.

## Materials and methods

### Animals and sampling

Rumen digesta samples were collected from five 10-month-old crossbred beef steers, which were raised under feedlot conditions on a high-energy finishing diet, as previously described (Hernandez-Sanabria et al., 2013) and followed the guidelines of the Canadian Council on Animal Care (Olfert et al., 1993). The animal protocol was approved by the Animal Care and Use Committee of University of Alberta (protocol no. Moore-2006-55). Animals were not starved before the sampling, and were slaughtered before feeding. For each animal, ~3 g of rumen digesta were collected at slaughter and stored in RNA*later* (Ambion, Carlsbad, CA, USA) at −20°C for further analysis.

### Nucleic acid extractions

Total RNA was extracted from rumen digesta using a modified procedure based on the acid guanidinium-phenol-chloroform method (Chomczynski and Sacchi, 1987; Béra-Maillet et al., 2009). Specifically, for ~200 mg of rumen digesta sample, 1.5 ml of TRIzol reagent (Invitrogen, Carlsbad, CA, USA), 0.4 ml of chloroform, 0.3 ml of isopropanol, and 0.3 ml of high salt solution (1.2 M sodium acetate, 0.8 M NaCl) were used. RNA quality and quantity was determined with the Agilent 2100 Bioanalyzer (Agilent Technologies, Santa Clara, CA, USA) and the Qubit 2.0 Fluorometer (Invitrogen, Carlsbad, CA, USA), respectively. RNA samples with the RNA integrity number (RIN) higher than 7.0 were used for downstream analysis. DNA was extracted from 25 to 30 mg of freeze-dried and ground rumen digesta according to the PCQI method (modified phenol-chloroform with bead beating and QIAquick PCR purification kit; Rius et al., 2012; Henderson et al., 2013).

### RNA library construction and sequencing (RNA-seq)

Total RNA (5 μl of 20 ng/μl) from each sample was used to construct an RNA library following the TruSeq RNA sample Prep v2 LS protocol (Illumina, San Diego, CA, USA), without the mRNA enrichment (rRNA removal) step. The quality and concentration of cDNA fragments (~260 bp) containing artificial sequences (adapters, index sequences, and primers; ~120 bp) and inserted cDNA sequences (~140 bp) were assessed using an Agilent 2100 Bioanalyzer (Agilent Technologies) and a Qubit 2.0 fluorometer (Invitrogen), respectively, before sequencing. RNA libraries were paired-end sequenced (2 × 100 bp) using an Illumina HiSeq2000 platform (McGill University and Génome Québec Innovation Centre, QC, Canada).

### Amplicon-seq of 16S rRNA/rDNA using pyrosequencing (RNA/DNA Amplicon-seq)

For the DNA Amplicon-seq, partial bacterial and archaeal 16S rRNA genes (the V1-V3 region for bacteria and the V6-V8 region for archaea) were amplified as previously described by Kittelmann et al. (2013) and sequenced using 454 GS FLX Titanium chemistry at Eurofins MWG Operon (Ebersberg, Germany). For the RNA Amplicon-seq, total RNA was first reverse-transcribed into cDNA using SuperScript II reverse transcriptase (Invitrogen) with random primers following procedures for first-strand cDNA synthesis. Then, partial 16S rRNA amplicons of bacteria and archaea were generated using the same primers as for the DNA Amplicon-seq and sequenced using a 454 pyrosequencing platform at McGill University and Génome Québec Innovation Centre (Montreal, QC, Canada).

### Analysis of the RNA-seq dataset

**Figure 1:**
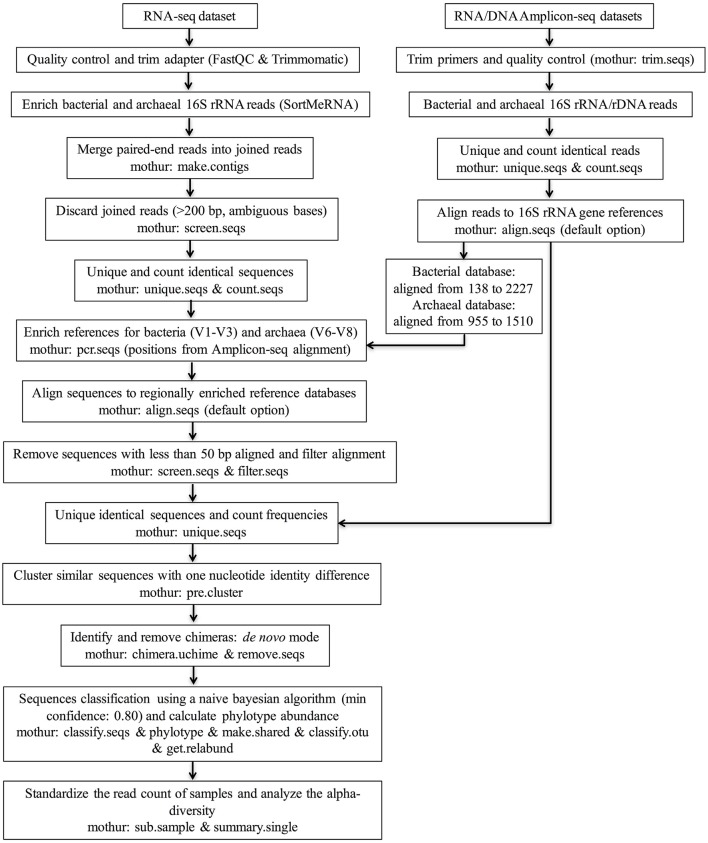
**Flow chart of the pipeline for analyzing rumen microbiota using RNA-seq and RNA/DNA Amplicon-seq**. The regionally enriched Greengenes 16S rRNA gene database (version gg_13_5_99 accessed May 2013) was used to analyze the bacterial community, and the regionally enriched rumen-specific archaeal database was used to analyze the archaeal community.

The sequence data quality was checked using the FastQC program (http://www.bioinformatics.babraham.ac.uk/projects/fastqc/). The program Trimmomatic (version 0.32; Bolger et al., 2014) was used to trim residual artificial sequences, cut bases with quality scores below 20, and remove reads shorter than 50 bp. The filtered reads were then sorted to enrich 16S rRNA fragments for taxonomic identification and mRNA reads for functional analysis (not reported in this study) using SortMeRNA (version 1.9; Kopylova et al., 2012) by aligning with the rRNA reference databases SILVA_SSU (release 119), SILVA_LSU (release 119; Quast et al., 2013), and Rfam 11.0 (Burge et al., 2013). After the 16S rRNA sequences were enriched, downstream analyses were performed using the mothur program (version 1.31.2; Schloss et al., 2009) according to the procedures (http://www.mothur.org/wiki/MiSeq_SOP) described by Kozich et al. (2013), with modifications. For taxonomic identification, regionally enriched reference datasets were built for bacteria and archaea (mothur command: pcr.seqs). Specifically, sequences belonging to the V1-V3 region (mean length: 466 bp) were extracted from the aligned Greengenes 16S rRNA gene database (version gg_13_5_99 accessed May 2013; DeSantis et al., 2006) for bacteria. For archaea, sequences belonging to the V6-V8 region (mean length: 456 bp) were extracted from the aligned rumen-specific archaeal 16S rRNA gene database derived from a previous study (Janssen and Kirs, 2008). The starting and ending positions of the targeted regions were located based on the alignment of Amplicon-seq reads to the references databases (Figure 1) because these amplicons were generated using designed primers for known regions.

The overall RNA-seq data analysis pipeline is illustrated in Figure 1. Briefly, sorted paired-end reads belonging to bacterial and archaeal 16S rRNA were joined (mothur command: make.contigs) to increase the length by combining the forward and reverse sequences. Joined sequences (mean length: 140 bp) with ambiguous bases or longer than 200 bp were discarded (mothur command: screen.seqs) to remove sequences without overlapped regions. Identical sequences were then binned to generate a set of unique sequences to facilitate the counting of their frequencies in each sample (mothur commands: unique.seqs and count.seqs). Next, the bacterial and archaeal 16S rRNA sequences in the sample datasets were aligned to the regionally enriched bacterial (the V1-V3 region) and archaeal (the V6-V8 region) references (see above), respectively, (mothur command: align.seqs with default option). Sequences for which less than 50 bp aligned to the reference datasets were culled (mothur command: screen.seqs). After alignment filtering (mothur command: filter.seqs), combining the identical sequences, and counting the frequencies (mothur command: unique.seqs), pre-clustering was performed to decrease the complexity of our sample datasets by clustering highly similar sequences with one nucleotide identity difference (mothur command: pre.cluster). UCHIME (Edgar et al., 2011) in *de novo* mode and with default settings was applied to identify and remove chimeras (mothur command: chimera.uchime & remove.seqs). Finally, chimera-depleted sequences were subject to taxonomic assignment to different phylotypes using a naive Bayesian algorithm (Wang et al., 2007) with a minimum confidence of 0.8 (mothur command: classify.seqs), and then the taxonomic rank and the relative abundance for each phylotype was calculated (mothur commands: phylotype, make.shared, classify.otu, and get.relabund). To make alpha-diversity estimators comparable among samples and different methods, all samples were standardized to the same number of sequences (the smallest sampling size) by randomly selecting sequences from the chimera-depleted datasets (mothur command: sub.sample). Alpha-diversity analysis was conducted at the bacterial family level and archaeal species level. The Good's coverage, the Shannon index, the inverse Simpson index, the number of observed phylotypes, and the Chao estimator were calculated based on the normalized samples (mothur command: summary.single).

### Analysis of RNA/DNA Amplicon-seq datasets

The procedures were similar to those described for the RNA-seq dataset (Figure 1). Briefly, after trimming primers and screening homopolymer runs (maximum length: 6), only sequences over 200 bp in length with an average quality score over 25 and with less than 6 ambiguous bases were included in the analysis. This step was performed using mothur (version 1.31.2; Schloss et al., 2009) with the command t*rim.seqs*. After clustering similar sequences, the chimeras were checked and discarded from reads with good quality. The chimera-depleted reads were used for taxonomic identification and to calculate the relative abundance of each phylotype. The alpha-diversity was analyzed using the standardized chimera-depleted sequences according to the lowest number of reads.

### Validation of microbial relative abundances using qRT-PCR and qPCR

Quantitative reverse transcription PCR (qRT-PCR) and quantitative PCR (qPCR) were further performed to validate the relative abundance data obtained from RNA-seq, RNA Amplicon-seq, and DNA Amplicon-seq. Three primer pairs (Supplementary Table 2) were used to enumerate the total bacteria, *Bacteroidetes*, and *Gammaproteobacteria* in each rumen sample. Standard curves were constructed using serial dilutions of plasmid DNA from clones identified as *Butyrivibrio hungatei* (for total bacteria, using an initial concentration of 8.50 × 10^8^ mol/μl), *Prevotella* sp. (for *Bacteroidetes*, using an initial concentration of 2.89 × 10^8^ mol/μl), and *Tolumonas auensis* (for *Gammaproteobacteria*, using an initial concentration of 2.68 × 10^8^ mol/μl). The copy numbers for each standard curve were calculated as described previously (Li et al., 2009). For qRT-PCR, cDNA was first reverse-transcribed from 20 ng of total RNA using iScript reverse transcription supermix for RT-qPCR (Bio-Rad, Hercules, CA, USA) and then diluted 5 times. One microliter of diluted cDNA was subjected to a qRT-PCR reaction using SYBR Green chemistry (Fast SYBRH Green Master Mix; Applied Biosystems) in a StepOnePlus Real-Time PCR System (Applied Biosystems). The relative abundances of *Bacteroidetes* and *Gammaproteobacteria* compared to total bacteria were calculated according to the following equation: relative abundance = Q_Target_/Q_U2_, where Q_Target_ was the quantity of each target, and Q_U2_ was the total bacteria quantity. Concurrently, we performed qPCR using total DNA (1 μl × 10 ng/μl total DNA per reaction) and followed the same procedures mentioned above to verify DNA Amplicon-seq results.

### Statistical analysis

In this study, only taxa with a relative abundance > 0.1% in at least two samples within the RNA-seq, RNA Amplicon-seq, and DNA Amplicon-seq datasets were defined as detectable and subjected to downstream statistical analysis. Statistical summaries (mean and SEM) of the detected taxa, and Pearson's correlation analysis for qRT-PCR/qPCR validation were all performed using R 3.1.2 (R Core Team, 2014). Principal coordinate analysis (PCoA) of the microbial profiles generated from the different datasets was conducted based on the Bray-Curtis dissimilarity matrix. The microbial relative abundance was arcsine-square-root transformed (Franzosa et al., 2014), and then Repeated Measures ANOVA was performed to compare the differences among three datasets. *P* values were adjusted into FDR using Benjamini-Hochberg method (Benjamini and Hochberg, 1995), and a threshold of FDR < 0.15 was applied to determine the significance (Korpela et al., 2016). Co-occurrence analysis was performed for the bacterial families and archaeal taxa detected in both the RNA-seq and DNA Amplicon-seq datasets (with relative abundance > 0.1% in all five samples) based on Spearman's rank correlation (Barberan et al., 2012). An association network was constructed using CoNet (Faust et al., 2012) and displayed using Cytoscape 3.2.1 (Shannon et al., 2003; Faust et al., 2012). Effective alpha-diversity estimators (Jost, 2007) were compared among the RNA-seq, RNA Amplicon-seq, and DNA Amplicon-seq datasets using paired Wilcoxon signed rank test.

### Data submission

RNA-seq and RNA Amplicon-seq datasets were submitted into the NCBI Sequence Read Archive (SRA) under the accession number PRJNA275012, and DNA Amplicon-seq sequences were also placed in the NCBI SRA under the accession number PRJNA273417.

## Results and discussion

### Analyzing rumen microbiota using RNA-seq and RNA/DNA Amplicon-seq

This study assessed active rumen microbial communities using RNA-seq and is the first study to compare RNA-seq outcomes with the well-accepted Amplicon-seq methods to evaluate rumen microbiota. It has been reported that rRNA levels directly relate to the protein synthesis potential of microorganisms (Blazewicz et al., 2013) and are correlated with activity (Poulsen et al., 1993; Bremer and Dennis, 2008). Therefore, rRNA abundance data obtained from total RNA sequencing could potentially be used as one of the indices to taxonomically assess potentially active microbes within a sample. To explore the possibility of taxonomic profiling using total RNA-seq, we firstly enriched 16S rRNA sequences from the RNA-seq dataset (Figure 1). In total, an average of 38,496,238 ± 2,037,011 (mean ± SEM) reads per sample (192,481,188 reads in total) were obtained after quality control filtration. Among them, 92.9 ± 1.1% belonged to small and large subunit rRNA, with 13.7 ± 5.6% and 0.2 ± 0.0% being bacterial and archaeal 16S rRNA, respectively, (Supplementary Table 1). It is notable that a large fraction of rRNA was classified as eukaryotic 18S (22.1 ± 5.9%) and 28S (32.2 ± 8.2%) rRNA (Supplementary Table 1). Although these reads were not analyzed in the current study, the high number of these sequences indicates the possibility of assessing rumen eukaryotic microbiota using RNA-seq in future studies. After combining paired-end reads and removing non-overlapping sections, 10,782,833 bacterial and 152,585 archaeal joint 16S rRNA sequences were obtained.

In this study, we included regionally enriched 16S rRNA gene reference datasets for taxonomic analysis rather than aligning the sequences to the full-length 16S rRNA gene database directly. Because the length of Illumina RNA-seq reads is short, 16S rRNA sequences could be randomly aligned to different regions of the 16S rRNA gene. It is known that different hypervariable regions of the 16S rRNA gene can affect diversity estimation and taxonomic classification (Logares et al., 2014). If these short 16S rRNA sequences are directly mapped to full-length 16S rRNA gene references for taxonomic analysis, as has been performed in previous studies (Urich et al., 2008; Tveit et al., 2014), it could lead to a mixed taxonomic profile as well as an overestimation of diversity. To avoid such potential bias, two regionally enriched reference datasets were generated from the Greengenes 16S rRNA gene database (version gg_13_5_99 accessed May 2013) (the V1-V3 region for bacteria) and the rumen-specific archaeal 16S rRNA database (Janssen and Kirs, 2008; the V6-V8 region for archaea; see details in the Materials and Methods Section). These regions were chosen because the V2-V3 region is the most efficient region for assessing bacterial community (Chakravorty et al., 2007), while the V6-V8 region is the most efficient region for identifying archaea and estimating the archaeal community diversity (Snelling et al., 2014). After identifying sequences belonging to the bacterial V1-V3 region and the archaeal V6-V8 region, sequences aligned with more than 50 bp of the reference datasets were then subjected to chimeric sequence detection (2,423,139 bacterial and 25,451 archaeal). After the removal of 9353 bacterial sequences (0.4%) and 139 archaeal sequences (0.6%) through chimera checking, 2,413,786 of the V1-V3 region-enriched bacterial sequences (mean length: 124 bp) and 25,312 of the V6-V8 region-enriched archaeal sequences (mean length: 133 bp) were subject to further taxonomic analysis (Table 1).

**Table 1:** **Summary of sequences used for taxonomic analysis from the chimera-depleted RNA-seq and Amplicon-seq datasets**.

	**RNA-seq**			**RNA Amplicon-seq**			**DNA Amplicon-seq**		
	**No. of reads**	**Classified (%)**	**Unclassified (%)**	**No. of reads**	**Classified (%)**	**Unclassified (%)**	**No. of reads**	**Classified (%)**	**Unclassified (%)**
Bacteria (phylum)	2,413,786	94.6	5.4	30,644	98.2	1.8	25,526	98.8	1.2
Bacteria (family)		86.9	13.1		86.8	13.2		87.1	12.9
Archaea (mixed)	25,312	98.0	2.0	7,741	98.6	1.4	6,041	99.7	0.3

RNA and DNA Amplicon-seq of the same rumen samples generated 37,105 (7421 ± 506; mean ± SEM; RNA Amplicon-Seq dataset) and 31,031 (6206 ± 645; DNA Amplicon-Seq dataset) bacterial reads, respectively, as well as 8303 (1661 ± 20 for RNA Amplicon-Seq) and 6663 (1333 ± 95 for DNA Amplicon-Seq) archaeal reads, respectively, (Supplementary Table 1). From these two datasets, 6461/5505 bacterial reads (17.4/17.7%) and 562/662 archaeal reads (6.8/9.3%) were detected and removed as chimeric sequences. In total, 30,644/25,526 bacterial reads (mean length: 476/487 bp) and 7741/6041 archaeal reads (mean length: 451/462 bp) were used for taxonomic identification and quantification (Table 1).

In this study, we used the *de novo* (database-independent) mode to determine chimeras rather than reference-based chimera detection. This is because the existing chimera reference databases only contain sequences from cultured organisms (Haas et al., 2011) and are not suitable for real samples that contain uncultured bacteria and archaea. Notably, higher percentages of chimeric sequences (17.4/17.7% of the bacterial and 6.8/9.3% of the archaeal sequences) were removed from the RNA/DNA Amplicon-seq datasets than from the RNA-seq dataset, which had only 0.4% of their bacterial and 0.6% of their archaeal sequences removed due to the presence of chimeras. In Amplicon-Seq datasets, chimeras are produced during PCR amplification, and they can lead to biased estimation of the diversity and/or the identification of differences between microbial communities (Edgar et al., 2011), while in RNA-seq datasets, chimeras might stem from the cDNA synthesis and/or fragment enrichment procedures used during the library construction. Our results indicate that RNA-seq was less affected by chimera formation than Amplicon-seq.

### Microbial taxa detected from RNA-seq and RNA/DNA Amplicon-seq

From the RNA-seq dataset, 94.6 and 86.9% of the bacterial V1-V3 region-enriched sequences were classified at the phylum and family level, respectively (Table 1). Due to their short sequence lengths, 86.2% of bacterial sequences from the RNA-seq dataset could not be classified at the genus level. Thus, only bacterial taxa at the phylum and family levels were retained for further analysis. For archaea, 98.0% of the V6-V8 region-enriched sequences were classified in a mixed taxonomic rank scheme (Table 1). We classified archaeal sequences at different taxonomic levels because most of the predominant archaeal phylotypes (such as *Methanobrevibacter ruminantium* and *Methanobrevibacter gottschalkii*) have well-studied 16S rRNA genes for use as references (Janssen and Kirs, 2008), and even short reads could be classified at the species level. However, for the poorly studied groups, such as *Methanomassiliicoccales*, reads could be only classified at the order level based on this rumen-specific archaeal database (Janssen and Kirs, 2008). From RNA/DNA Amplicon-seq datasets, 98.2/98.8%, 86.8/87.1%, and 98.6/99.7% of the total reads were assigned at the bacterial phylum, bacterial family and archaeal mixed taxa levels, respectively (Table 1).

**Figure 2:**
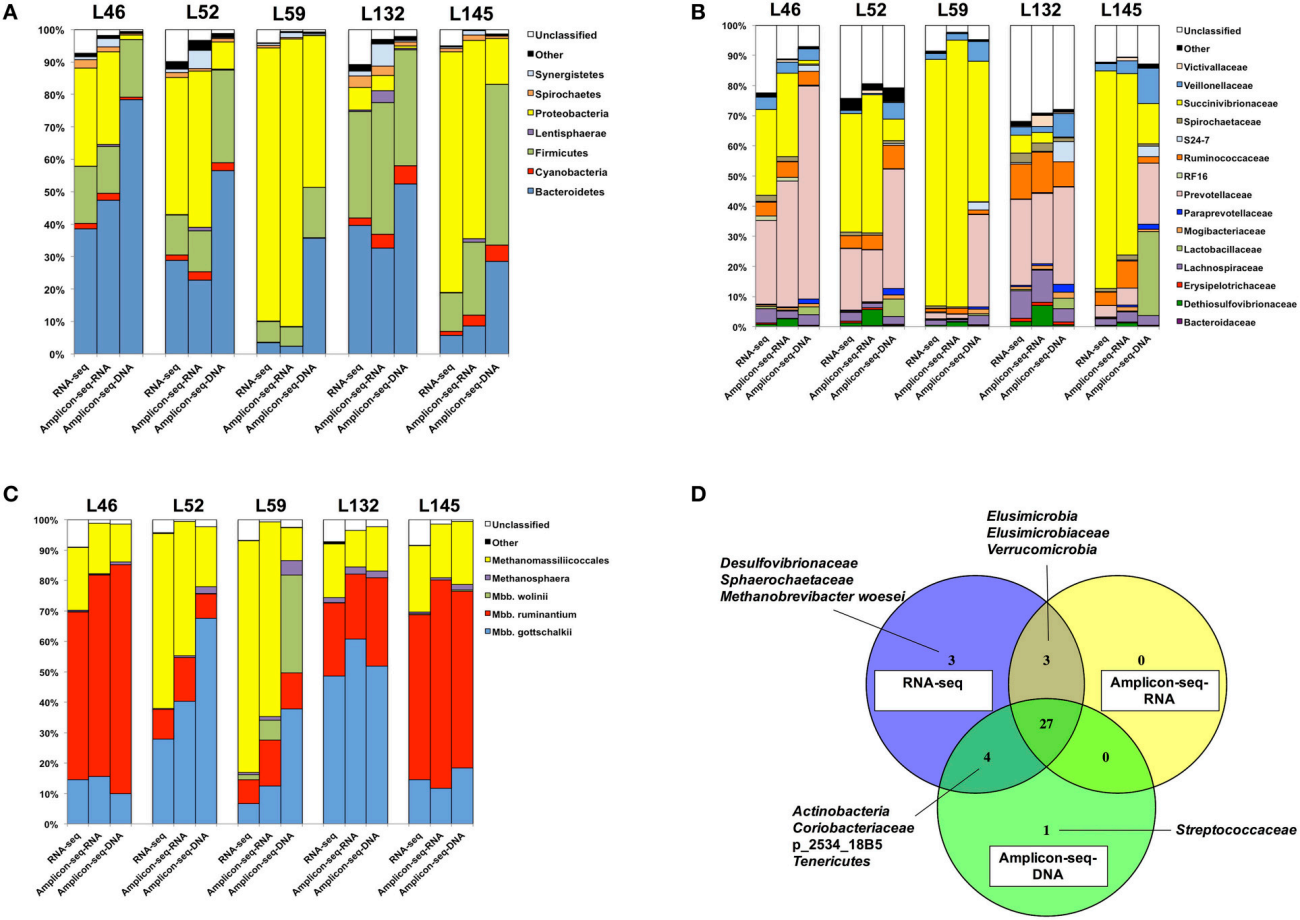
**Microbial community composition estimated in the RNA-seq and RNA/DNA Amplicon-seq datasets**. Microbial community composition of **(A)** bacterial phyla, **(B)** bacterial families, **(C)** archaea, and **(D)** dataset-specific taxa.

The bacterial and archaeal taxa detected in the three datasets were generally similar, with a total of 11 bacterial phyla, 21 bacterial families and six archaeal taxa identified. Of these, seven bacterial phyla, 15 bacterial families and five archaeal taxa were commonly detected across the three datasets (Figure 2). Notably, there were unique bacterial and archaeal taxa identified in each dataset (Figure 2D). Firstly, two bacterial families and one archaeal taxon (*Desulfovibrionaceae, Sphaerochaetaceae*, and *Methanobrevibacter woesei*) were detected only in the RNA-seq dataset and not in the RNA/DNA Amplicon-seq datasets. Henderson et al. (2015) also reported the absence of *Desulfovibrionaceae* and *Methanobrevibacter woesei*, and the low abundance (≤ 0.1%) of *Sphaerochaetaceae* in the rumen digesta when using DNA Amplicon-seq with the same PCR primers, suggesting that the amplicon-based approach and/or the primers used may have masked the detection of these taxa in the rumen. In addition, lower sequencing depth of RNA/DNA Amplicon-seq could also lead to the missing detection of these taxa. As shown in Table 1, the number of bacterial and archaeal reads from the RNA-seq dataset was about 80–90 times higher and 3–4 times higher than that from RNA/DNA Amplicon-seq datasets, respectively. And thus, increasing sequencing depth could probably enhance the detection of these taxa in the Amplicon-seq datasets. Secondly, two bacterial phyla (*Elusimicrobia* and *Verrucomicrobia*) and one bacterial family (*Elusimicrobiaceae*) were detected only in the RNA-based datasets (RNA-seq and RNA Amplicon-seq) and not in the DNA Amplicon-seq dataset. Our results suggest that these two phyla may be more active in the rumen, and they may be underestimated based on the DNA Amplicon-seq dataset. Moreover, the absence of *Elusimicrobia* and *Verrucomicrobia* in the DNA Amplicon-seq dataset may be due to the unsuccessful isolation of their DNA, and it has been reported that various DNA extraction methods could impact the taxonomic outcomes of rumen microbiota assessments (Henderson et al., 2013). Finally, the bacterial family *Streptococcaceae* was detected only in the DNA Amplicon-seq dataset with a low relative abundance of 0.1 ± 0.1%. Previous studies on the bacterial profiles of rumen digesta from the same cattle used in this study (Hernandez-Sanabria et al., 2012) and different cattle (Petri et al., 2013; Xia et al., 2015) have also reported the absence of *Streptococcaceae*. In a recent study based on a large number (*n* = 742) of rumen and foregut digesta samples and DNA-based Amplicon-seq, *Streptococcaceae* also showed low prevalence in all animals (Henderson et al., 2015). These suggest that *Streptococcaceae* may have low cellular abundance and even lower activities in samples assessed in the current study.

### Microbial relative abundances estimated from RNA-seq and RNA/DNA Amplicon-seq

**Figure 3:**
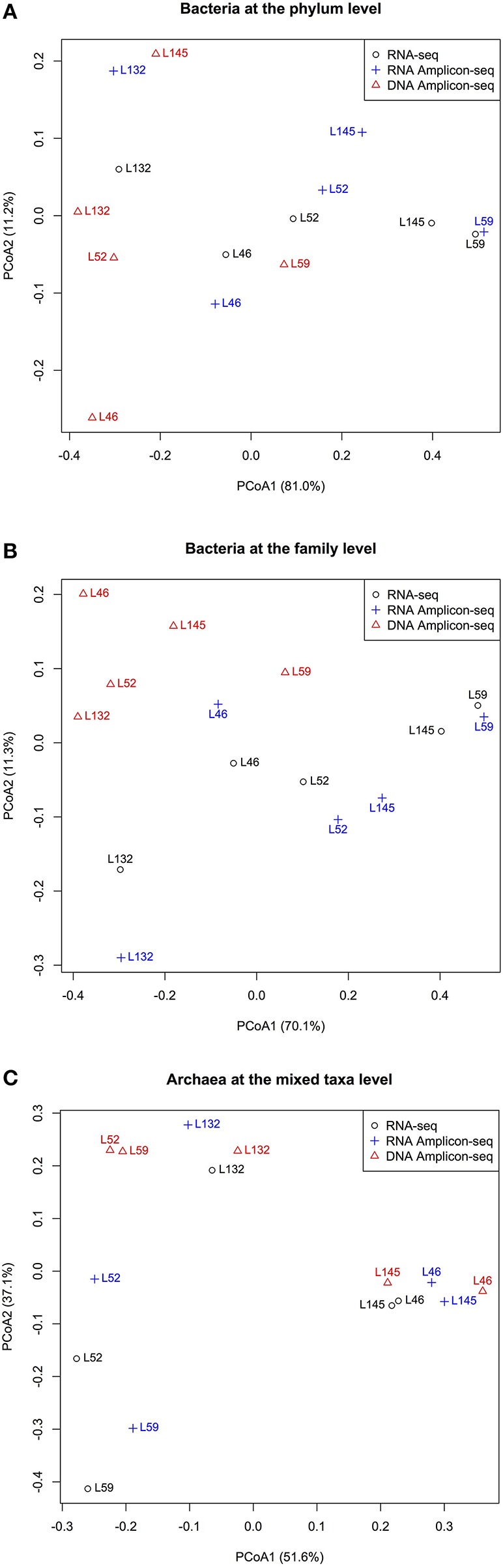
**Dissimilarities among the RNA-seq, RNA Amplicon-seq and DNA Amplicon-seq datasets revealed by principal coordinate analysis (PCoA). (A)** PCoA based on shared bacterial phyla, **(B)** PCoA based on shared bacterial families, **(C)** PCoA based on shared archaeal mixed taxa. PCoA was performed based on the Bray-Curtis dissimilarity matrix.

Principal coordinate analysis (PCoA) of the relative abundances of commonly detected bacterial phyla, bacterial families, and archaeal taxa revealed dissimilarities in microbial profiles between RNA- and DNA-based approaches (Figure 3). For each animal, the RNA-seq and RNA Amplicon-seq generated similar rumen bacterial profiles (at both the phylum and family levels), which generally separated with that assessed using DNA Amplicon-seq (Figures 3A,B). However, for each animal, three datasets displayed generally similar archaeal profiles (Figure 3C); the RNA-based assessment outcomes of L52 and L59 were distinct from their DNA-based profiles because these two samples had a high abundance of *Methanomassiliicoccales* in the RNA-based datasets (Figure 2C). Among the shared taxa, four bacterial phyla (*Bacteroidetes, Lentisphaerae, Proteobacteria*, and *Synergistetes*), eight bacterial families (*Dethiosulfovibrionaceae, Lactobacillaceae, Mogibacteriaceae, Paraprevotellaceae, Prevotellaceae*, S24-7, *Succinivibrionaceae*, and *Victivallaceae*), and one archaeal taxon (*Methanomassiliicoccales*) had significantly different relative abundances among the three datasets (FDR < 0.15; Table 2). Lanzen et al. (2011) reported that the dominant taxa within hydrothermal vent field microbiota showed similar outcomes on the RNA and DNA levels based on their Amplicon-seq results, which is not consistent with our findings and is probably an extremely environment-specific case. In addition, such discrepancy may also be due to different targeted amplicon regions (the V5-V6 region in Lanzen et al. (2011) vs. the V1-V3/V6-V8 regions in the current study) as well as different reference databases used (the Silva SSURef in Lanzen et al. (2011) vs. the regionally enriched Greengenes/rumen-specific archaeal databases in our study). Therefore, different methods and strategies should be carefully considered for samples from various environmental conditions.

**Table 2:** **Differential taxa among the RNA-seq and RNA/DNA Amplicon-seq datasets^1^**.

**Taxonomy level**	**Classification**	**RNA-seq (%) (Mean ± SEM)**	**RNA Amplicon-seq (%)**	**DNA Amplicon-seq (%)**	**FDR^2^**
**BACTERIA**					
Phyla	*Bacteroidetes*	23.3 ± 7.9^a^	22.7 ± 8.1^a^	50.3 ± 8.7^b^	0.09
Family	*Paraprevotellaceae*	0.2 ± 0.1^a^	0.3 ± 0.1^a^	1.8 ± 0.3^b^	0.09
Family	*Prevotellaceae*	16.4 ± 5.7^a^	17.9 ± 7.2^a^	38.7 ± 8.6^b^	0.14
Family	S24-7	0.2 ± 0.1^a^	0.2 ± 0.1^a^	3.1 ± 1.0^b^	0.09
Phyl^a^	*Firmicutes*^3^	16.2 ± 4.5	19.2 ± 6.0	29.4 ± 6.2	0.35
Family	*Lactobacillaceae*	0.3 ± 0.1^a^	0.2 ± 0.1^a^	8.1 ± 5.0^b^	0.09
Family	*Mogibacteriaceae*	0.5 ± 0.1^a^	0.8 ± 0.2a^b^	1.3 ± 0.2^b^	0.09
Phyl^a^	*Lentisphaerae*	0.2 ± 0.1^a^	1.3 ± 0.6^b^	0.1 ± 0.1^a^	0.09
Family	*Victivallaceae*	0.2 ± 0.1^a^	1.3 ± 0.6^b^	0.1 ± 0.1^a^	0.09
Phyl^a^	*Proteobacteria*	47.6 ± 14.2^a^	46.3 ± 14.3^a^	14.3 ± 8.5^b^	0.09
Family	*Succinivibrionaceae*	45.6 ± 14.0^a^	45.1 ± 14.4^a^	13.8 ± 8.6^b^	0.09
Phyl^a^	*Synergistetes*	0.9 ± 0.2^a^	3.6 ± 1.1^b^	0.3 ± 0.1c	0.12
Family	*Dethiosulfovibrionaceae*	0.8 ± 0.2^a^	3.6 ± 1.1^b^	0.3 ± 0.1c	0.12
**ARCHAEA**					
Mixed	*Methanomassiliicoccales*	38.8 ± 11.9^a^	30.9 ± 10.0^b^	15.7 ± 2.0a^b^	0.09

1 *Only commonly detected phylotypes among three datasets were compared*.

2 *P values were obtained using Repeated Measures ANOVA based on the arcsine-square-root transformed proportion values, and then were adjusted into FDR using Benjamini-Hochberg method (Benjamini and Hochberg, 1995). A threshold of FDR < 0.15 was applied to determine the significance. Within a row, means with different superscript are significantly different*.

3 *The relative abundance of the bacterial phylum Firmicutes did not show differences, but two families belonged to this phylum were different among datasets*.

The dominant bacterial phyla detected by DNA Amplicon-seq were *Bacteroidetes* (50.3 ± 8.7%; mean ± SEM), *Firmicutes* (29.4 ± 6.2%), and *Proteobacteria* (14.3 ± 8.5%), which is consistent with previous studies using DNA-based methods. For example, *Bacteroidetes* (range, 8.0–60.1%) and *Firmicutes* (range, 33.6–85.0%) were reported as the most abundant phyla, and *Proteobacteria* was commonly detected but less abundant (range, 0.6–20.1%) in the rumen (Jami and Mizrahi, 2012; Petri et al., 2013; Kim and Yu, 2014). However, the predominant bacterial phylum detected by RNA-based approaches (RNA-seq and RNA Amplicon-seq) was *Proteobacteria* (47.6 ± 14.2 and 46.3 ± 14.3%, respectively), followed by *Bacteroidetes* (23.3 ± 7.9 and 22.7 ± 8.1%), and *Firmicutes* (16.2 ± 4.5 and 19.2 ± 6.0%). The higher proportion of *Proteobacteria* in the RNA-based datasets confirmed similar findings by Kang et al. (2013) and Kang et al. (2009), who applied RNA amplicon-based sequencing and rRNA-based clone libraries, respectively, to study the rumen microbiota. At the bacterial family level, the most abundant bacterial family was *Succinivibrionaceae* (belonging to the phylum *Proteobacteria*) in the RNA-seq (45.6 ± 14.0%) and RNA Amplicon-seq (45.1 ± 14.4%) datasets, while it was *Prevotellaceae* (belonging to the *Bacteroidetes*) in the DNA Amplicon-seq dataset (38.7 ± 8.6%). *Succinivibrionaceae* was an abundant family at the DNA level when ruminants were fed high-energy diets (Hernandez-Sanabria et al., 2012; Petri et al., 2013; Henderson et al., 2015), and the significance of *Succinivibrionaceae* may be underestimated using DNA Amplicon-seq. The predominance of *Prevotellaceae* detected in the DNA Amplicon-seq dataset is similar to that observed in previously studies using DNA-based approaches (Kittelmann et al., 2013; Petri et al., 2013; Henderson et al., 2015). However, its abundance, as estimated in the RNA-seq (16.4 ± 5.7%) and RNA Amplicon-seq (17.9 ± 7.2%) datasets, was significantly lower (FDR < 0.15). The family *Lactobacillaceae*, belonging to the phylum *Firmicutes*, also had a lower abundance in the RNA-based datasets (0.3 ± 0.1% using RNA-seq and 0.2 ± 0.1% using RNA Amplicon-seq) than in the DNA Amplicon-seq dataset (8.1 ± 5.0%, FDR < 0.15). These two families probably had higher cellular abundances but relatively lower activities in the bovine rumen.

From the RNA/DNA Amplicon-seq datasets, *Methanobrevibacter gottschalkii* (28.2 ± 9.7 /37.1 ± 10.6%), *Methanobrevibacter ruminantium* (37.1 ± 12.4/36.4 ± 13.1%), and *Methanomassiliicoccales* (30.9 ± 10.0/15.7 ± 2.0%) were dominant but with different rankings. The DNA Amplicon-seq outcomes are generally consistent with previous studies that used the same approaches (Kittelmann et al., 2013; Henderson et al., 2015). The archaeal taxon *Methanomassiliicoccales*, which has been previously referred to as Rumen Cluster C or *Thermoplasmatales* (Janssen and Kirs, 2008; Poulsen et al., 2013; Gaci et al., 2014), was predominant in the RNA-seq dataset (38.8 ± 11.9%), followed by *Methanobrevibacter ruminantium* (30.2 ± 10.4%), and *Methanobrevibacter gottschalkii* (22.4 ± 7.4%). The high proportion of *Methanomassiliicoccales* from the RNA-based datasets supports the hypothesis that they are more active in the rumen, as many studies have suggested (Ohene-Adjei et al., 2007; Wright et al., 2007; Janssen and Kirs, 2008; Williams et al., 2009; Jeyanathan et al., 2011).

### qRT-PCR and qPCR validation of microbial relative abundances among datasets

qRT-PCR and qPCR were performed to estimate the relative abundances of two predominant phyla (*Bacteroidetes* and *Proteobacteria*) among all three datasets. *Gammaproteobacteria* was selected to represent *Proteobacteria* because 95.8, 98.0, and 96.5% of the *Proteobacteria* reads from the three datasets belonged to the class *Gammaproteobacteria*. The qRT-PCR results for *Bacteroidetes* were in agreement with the RNA-seq (Pearson's correlation coefficient [*r*] = 0.97, *P* < 0.05) and the RNA Amplicon-seq (*r* = 0.97, *P* < 0.05) results, and the qPCR results for *Bacteroidetes* were also consistent with the DNA Amplicon-seq results (*r* = 0.88, *P* = 0.05; Supplementary Figure S1). The relative abundance of *Gammaproteobacteria* estimated using qRT-PCR was correlated with that from the RNA-seq dataset (*r* = 0.97, *P* < 0.05) and the RNA Amplicon-seq dataset (*r* = 0.99, *P* < 0.05), and there was also a high degree of correlation between qPCR and DNA Amplicon-seq (*r* = 0.99, *P* < 0.05) for *Gammaproteobacteria*. The overall consistent trends between the RNA-based approaches and qRT-PCR (as well as between DNA Amplicon-seq and qPCR) confirm the different relative abundance detected in the three datasets.

### Alpha-diversity estimators in the RNA-seq and RNA/DNA Amplicon-seq datasets

In the present study, alpha-diversity indices were estimated based on the observed phylotypes at the family level for bacteria and at the species level for archaea. To avoid potential differences caused by different sequencing depths among the three datasets, the samples were randomly normalized according to the lowest number of reads (3476 bacterial sequences and 1074 archaeal sequences per sample after normalization). The values of Good's coverage were all above 99% for the bacterial and archaeal data from the three datasets, indicating that the numbers of reads after normalization were sufficient to represent the microbial communities. The Shannon index and the inverse Simpson index were not significantly different (*P* > 0.1, the paired Wilcoxon signed rank test) among the three datasets (Table 3). The number of observed phylotypes and the Chao estimator tended to be higher in the RNA-seq dataset than in the RNA/DNA Amplicon-seq datasets for bacteria and archaea (*P* < 0.1, the paired Wilcoxon signed rank test; Table 3), which was further confirmed using rarefaction analysis (Supplementary Figure S2). These results suggest that more microbial taxa could be detected using RNA-seq than using RNA/DNA Amplicon-seq. In the Amplicon-seq datasets, some phylotypes were probably overlooked due to the bias of primers and/or amplification conditions during the PCR process, which may explain the difference in richness among the three datasets.

**Table 3:** **A comparison of alpha-diversity estimators among the RNA-seq and RNA/DNA Amplicon-seq datasets**.

	**Bacteria**			**Archaea**		
	**RNA-seq**	**RNA Amplicon-seq (Mean ± SEM)**	**DNA Amplicon-seq**	**RNA-seq**	**RNA Amplicon-seq (Mean ± SEM)**	**DNA Amplicon-seq**
Number of observed phylotypes	45.4 ± 2.8^*^^1^	33.2 ± 2.9^#^	35.6 ± 3.1^#^	11.8 ± 0.4^a^	6.2 ± 0.2^b^	7.6 ± 0.5^c^
Chao	52.1 ± 3.8^*^	43.0 ± 7.3^*^^#^	40.4 ± 3.3^#^	12.7 ± 0.5^a^	6.2 ± 0.2^b^	8.4 ± 1.0^b^
Shannon^2^	1.9 ± 0.3	1.8 ± 0.3	2.0 ± 0.2	1.2 ± 0.1	1.0 ± 0.0	1.1 ± 0.1
Inverse Simpson	4.3 ± 1.2	4.2 ± 1.3	4.8 ± 1.0	2.5 ± 0.2	2.3 ± 0.1	2.5 ± 0.3
Good's coverage	99.7%	99.8%	99.8%	99.8%	100%	99.9%

1 *Within a row, means with different superscript tend to be different at P < 0.1. Comparison was conducted using paired Wilcoxon signed rank test for bacterial and archaeal communities separately, and thus estimators between bacterial and archaeal groups are not comparable*.

2 *Shannon indices showed in the table are the raw values, and the comparison of Shannon indices among datasets was based on the exponentially transformed values (Jost, 2007) using paired Wilcoxon signed rank test*.

### Co-occurrence analysis of abundant microbial taxa detected from RNA-seq and DNA Amplicon-seq

**Figure 4:**
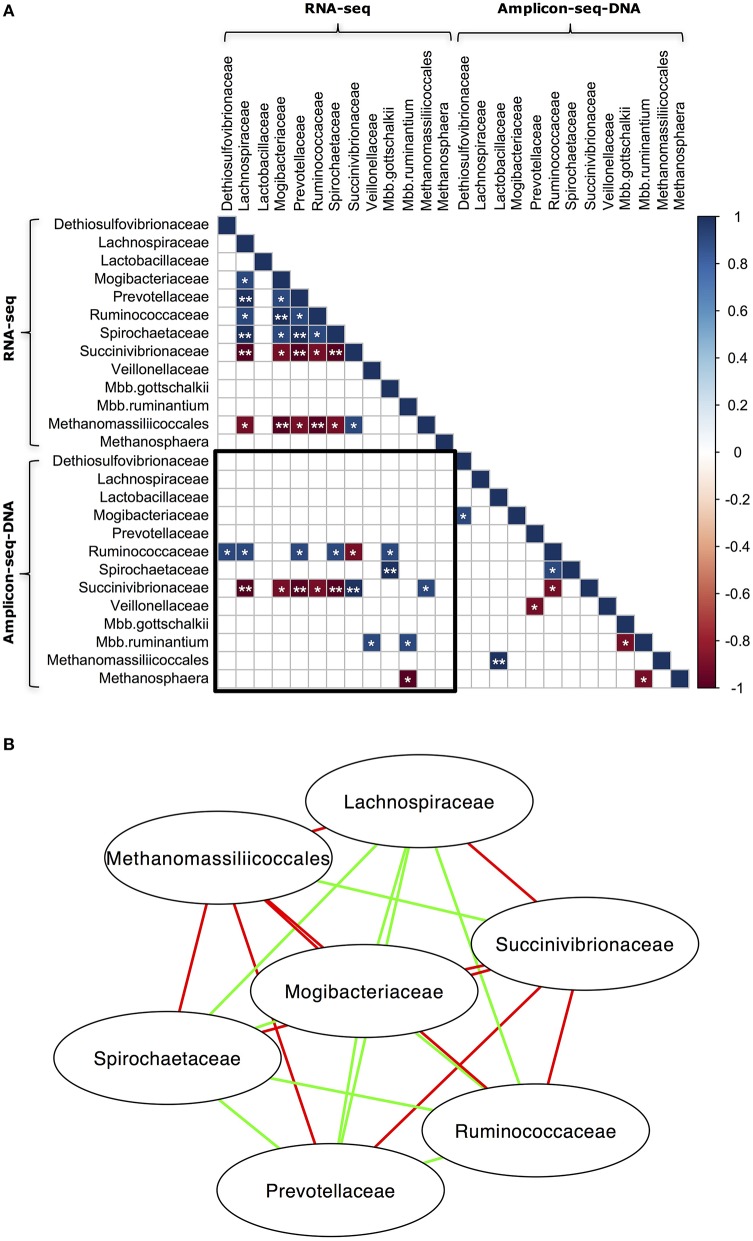
**Co-occurrence of abundant microbial taxa in the RNA-seq and DNA Amplicon-seq datasets. (A)** Correlation matrix of abundant microbial taxa and **(B)** Network of abundant microbial taxa in the RNA-seq dataset. Only bacterial families and archeael taxa with a relative abundance > 0.1% and detected in all five rumen samples using both RNA-seq and DNA Amplicon-seq were analyzed using Spearman's rank correlation. The RNA Amplicon-seq dataset was not included into the analysis, because its bacterial and archaeal profiles were similar to profiles from the RNA-seq dataset. In **(A)**, the sub-matrix surrounded by the black square exhibits correlations between taxa in the RNA-seq and DNA Amplicon-seq datasets. Strong correlations (Spearman's rank correlation coefficient [ρ] ≥ 0.9 or ≤ −0.9) were displayed with ^*^ (0.05 < *P* < 0.1) and ^**^ (*P* < 0.05), while the other correlations were showed as blank. In **(B)**, a connection with a green/red line means a strong positive/negative correlation (ρ≥0.9 or ≤ −0.9 and *P* < 0.1).

The relationships that exist among microbial taxa could be a determining factor for microbial community composition (Prosser et al., 2007). To explore the relationships among different taxa in our samples, Spearman's rank correlation was used to identify the co-occurrence patterns of different microbial groups in both the RNA-seq and DNA Amplicon-seq datasets (Figure 4). The RNA Amplicon-seq dataset was not included due to its similar outcomes to the RNA-seq dataset, with high correlation for all samples (Spearman's rank correlation coefficient [ρ] = 0.88 − 0.98, *P* < 0.0001; Figures 2, 3). As shown in Figure 4, the microbial taxa identified using RNA-seq were more closely correlated than those identified using DNA Amplicon-seq. There may be stronger interactions among microbes within a microbiota at the transcriptional level than that at the genomic level, which has been suggested recently by İnceoǧlu et al. (2015). Previous studies have revealed associations among microbes (*Ruminococcaceae* and *Mbb. gottschalkii, Succinivibrionaceae* and *Methanomassiliicoccales, Mbb. gottschalkii* and *Mbb. ruminantium*, and *Methanomassiliicoccales* and *Methanosphaera*, etc) in rumen using DNA Amplicon-seq (Kittelmann et al., 2013; Henderson et al., 2015). In this study, we also detected a negative relationship between *Mbb. gottschalkii* and *Mbb. ruminantium* (ρ = −0.9, *P* < 0.1; Figure 4) in the DNA Amplicon-seq dataset. In the RNA-seq dataset, we confirmed that *Succinivibrionaceae* was positively correlated with *Methanomassiliicoccales* (ρ = 0.9, *P* < 0.1; Figure 4). The bacterial families *Lachnospiraceae, Mogibacteriaceae, Prevotellaceae, Ruminococcaceae*, and *Spirochaetaceae* were positively correlated with each other (*P* < 0.1), and all of them were negatively correlated with *Succinivibrionaceae* and *Methanomassiliicoccales* (*P* < 0.1) in the RNA-seq dataset (Figure 4B). However, the negative correlations between *Succinivibrionaceae* and the other 4 bacterial families, and between *Mbb. gottschalkii* and *Mbb. ruminantium*, are possibly because they displayed the arithmetic replacement effect (shifts in abundance of predominant phylotypes will have effects on others when analyzing proportion data) as suggested by Henderson et al. (2015). Meanwhile, only two taxa, *Succinivibrionaceae* and *Mbb. ruminantium*, showed significantly positive correlations between the RNA-seq and DNA Amplicon-seq datasets (ρ = 1.0, *P* < 0.05 and ρ = 0.9, *P* < 0.1, respectively). No other taxa exhibited strong consistency between the RNA-seq and DNA Amplicon-seq datasets, indicating that cellular abundance did not correspond to the activities of most rumen taxa.

### Methodological caveats of this study

This study has limitations that should be taken into account. Firstly, the sampling timing may have more of an effect on RNA-based than DNA-based approaches of profiling microbial communities. If assessing the activities of rumen microbiota is the main study objective, the sampling timing should be carefully considered. In this study, rumen digesta samples were collected before the feeding, which probably resulted in different RNA profiles than in digesta samples collected after feeding. However, the same rumen digesta sample was used for RNA and DNA extraction in our study, so the detected differences between RNA- and DNA-based analyses are valid and not biased due to different sampling times. Second, during RNA isolation processes, RNA yield may differ according to extraction method (such as between physical, mechanic, enzymatic, and chemical methods; Stark et al., 2014). Meanwhile, not all microbes can be lysed with equal efficiency, and notably, RNA yields from Gram-positive bacteria are generally lower than those from Gram-negative bacteria (Stark et al., 2014). For instance, members of *Proteobacteria* and *Bacteroidetes* are Gram-negative, while most *Firmicutes* members in the rumen are Gram-positive, which may explain the higher abundances of *Proteobacteria* and *Bacteroidetes*, and the lower abundance of *Firmicutes* in the RNA-seq dataset. Moreover, significant differences in microbial community structures were also demonstrated to correspond to different DNA extraction methods in a report by Henderson et al. (2013). Third, because methods (Griffiths et al., 2000; Leininger et al., 2006) for co-extraction of RNA and DNA could not generate the high quality RNA for RNA-seq of our samples (RNA integrity number < 3.0), the RNA and DNA extractions were conducted separately using two independent protocols to ensure the high quality RNA and DNA in the current study, which could potentially lead to differences between RNA- and DNA-based methods. Furthermore, the RNA was transcribed to cDNA using the random primers before making the RNA-seq and RNA Amplicon-seq libraries, while the DNA Amplicon-seq was performed using region-specific primers to amplify DNA template directly, which could also contribute to differences among three datasets. Fourth, because rRNA content per cell varies between different microbial phylotypes (Medlin and Simon, 1998; Sievert et al., 2000), such intrinsic differences in rRNA content could also influence the relative abundance determined using RNA-seq. Future experiments to globally compare the rRNA content per cell among different microbial phylotypes in the rumen microbiota and normalize rRNA concentrations from different phylotypes can improve the accuracy of microbial community profiling using RNA-seq. Fifth, the quantification of total and/or species-specific rRNA is a valid and well-accepted approach to estimate the microbial activity, which has been applied in more than 100 studies (Blazewicz et al., 2013). However, the use of rRNA as an indicator of specific microbial functional activity in complex environmental samples still needs to be further validated by correlating them with the mRNA information within the same RNA-seq dataset. Sixth, in the current study, the RNA-seq dataset was generated on an Illumina HiSeq2000 platform, whereas RNA/DNA Amplicon-seq was performed using a 454 pyrosequencing platform. It has been demonstrated that there are different features between these two platforms, such as read length, accuracy, and throughput (Liu et al., 2012). However, previous studies have revealed the consistency of microbial community profiles generated across sequencing platforms (Caporaso et al., 2012; Nelson et al., 2014), but differences in sequencing depth could have an impact on the detection of low-abundant taxa.

## Conclusion

A comparison of the microbial profiles generated from RNA-seq and RNA/DNA Amplicon-seq revealed the generation of different taxonomic profiles of the same rumen microbiota between these methods, and thus, their results could not be simply combined. The RNA-based methods could more robustly detect microbial phylotypes with potentially metabolic activities in the rumen and also detect more interactions among these phylotypes than DNA Amplicon-seq. In addition, compared to RNA/DNA Amplicon-seq, the RNA-seq approach showed more diversity and could detect more bacterial and archaeal phylotypes in the rumen. Although the RNA-seq approach has the advantage of simultaneously identifying and quantifying active microorganisms within a microbiota, the data are not conclusive on which method is the best for analyzing animal gastrointestinal microbiota due to the different technologies and constraints of DNA vs. RNA and due to differences in nucleotide extraction, sequencing, and analysis protocols. Nevertheless, this is the first study to compare RNA-seq and RNA/DNA Amplicon-seq for the taxonomic assessment of rumen microbiota, and differences among these methods should be carefully considered to accurately assess gastrointestinal microbiota in future studies.

## Author contributions

FL: experimental design, data generation, data analysis and interpretation, manuscript writing; GH: DNA Amplicon-seq, data interpretation and manuscript writing; XS: protocol optimization of RNA-seq library construction; FC: DNA isolation and DNA Amplicon-seq; PHJ: data interpretation and manuscript writing; LLG: experimental design, data analysis and interpretation, manuscript writing.

### Conflict of interest statement

The authors declare that the research was conducted in the absence of any commercial or financial relationships that could be construed as a potential conflict of interest.
