# Supplementary material for: Taxonomic Assessment of Rumen Microbiota Using Total RNA and Targeted Amplicon Sequencing Approaches
Source: Front Microbiol. 2016 Jun 22;7:987. doi: 10.3389/fmicb.2016.00987 (PMC4916217; doi:10.3389/fmicb.2016.00987)
Supplement: Supplementary file 1 [file Presentation1.PDF]

Supplementary Material

Taxonomic assessment of rumen microbiota using total RNA and targeted amplicon sequencing approaches

Fuyong Li[1], Gemma Henderson[2], Xu Sun[1], Faith Cox[2], Peter H. Janssen[2], Le Luo Guan[1]*

[1]Department of Agricultural, Food and Nutritional Science, University of Alberta, Edmonton, Alberta, Canada

[2]AgResearch Ltd, Grasslands Research Centre, Palmerston North, New Zealand

*Correspondence:

Le Luo Guan

Email: lguan@ualberta.ca

SUPPLEMENTARY TABLE 1 General sequence profiles of RNA-seq[1] and RNA/DNA Amplicon-seq[2] datasets.

Method		Sample					Mean ± SEM
		L46	L52	L59	L132	L145	
RNA-seq	Bacterial 16S rRNA (%)	4.2	4.5	30.0	5.3	24.5	13.7±5.6
	Bacterial 23S rRNA (%)	7.7	9.3	50.6	9.4	42.5	23.9±9.3
	Archaeal 16S rRNA (%)	0.1	0.1	0.3	0.2	0.4	0.2±0.0
	Archaeal 23S rRNA (%)	0.4	0.2	0.8	0.9	1.5	0.7±0.2
	Eukaryotic 18S rRNA (%)	30.9	30.3	5.5	33.5	10.0	22.1±5.9
	Eukaryotic 28S rRNA (%)	47.8	46.3	9.2	42.3	15.5	32.2±8.2
	5S rRNA (%)	0.0	0.0	0.0	0.0	0.0	0.0±0.0
	5.8S rRNA (%)	0.1	0.1	0.0	0.1	0.0	0.1±0.0
	Other RNA (%)	8.8	9.2	3.7	8.4	5.6	7.1±1.1
	No. of total reads	42,161,316	41,860,772	41,422,056	33,763,140	33,273,904	38,496,238±2,037,011
RNA Amplicon-seq	No. of bacterial reads	7,331	8,513	8,472	6,991	5,798	7,421±506
	No. of archaeal reads	1,671	1,698	1,707	1,616	1,611	1,661±20
DNA Amplicon-seq	No. of bacterial reads	5,690	5,262	7,932	4,632	7,515	6,206±645
	No. of archaeal reads	1,157	1,177	1,684	1,286	1,359	1,333±95

1. RNA-seq: total RNA sequencing.

2. Amplicon-seq: sequencing of targeted PCR amplicons of bacterial and archaeal 16S rRNA/rDNA.

SUPPLEMENTARY TABLE 2 qRT-PCR and qPCR primers used in this study.

Target		Sequence (5'->3')	Reference
Total Bacteria	Forward	ACTCCTACGGGAGGCAG	(Stevenson and
	Reverse	GACTACCAGGGTATCTAATCC	Weimer, 2007)
Phylum *Bacteroidetes*	Forward	CAGCAGCCGCGGTAATAC	(Schwieger and
	Reverse	CCGTCAATTCCTTTGAGTTT	Tebbe, 1998)
Class *Gammaproteobacteria*	Forward	CMATGCCGCGTGTGTGAA	(Muhling et al.,
	Reverse	ACTCCCCAGGCGGTCDACTTA	2008)

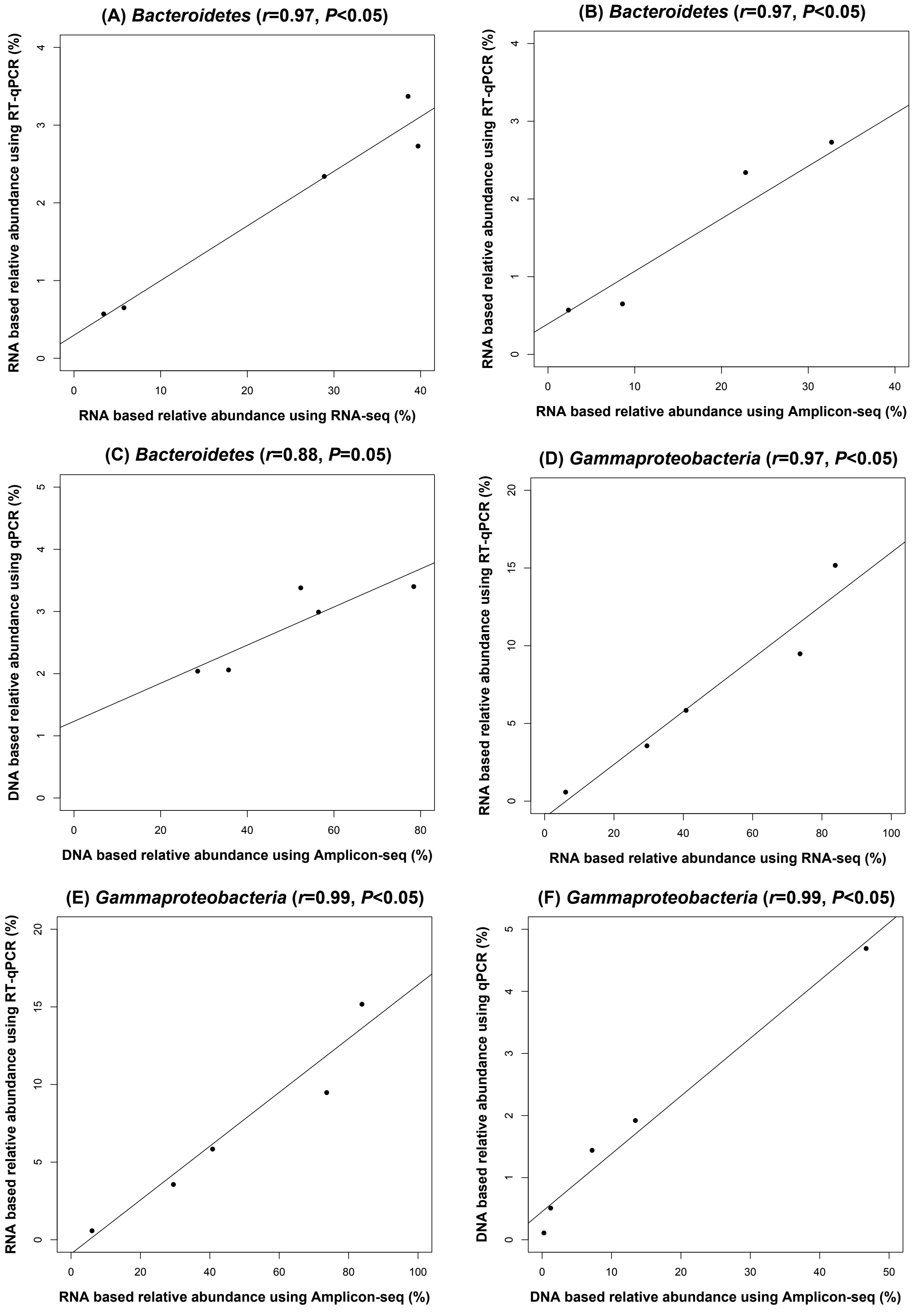

SUPPLEMENTARY FIGURE S1 Validation of bacterial relative abundances using qRT-PCR and qPCR. Relative abundances of *Bacteroidetes* (A) and *Gammaproteobacteria* (D) from qRT-PCR and RNA-seq dataset, *Bacteroidetes* (B) and *Gammaproteobacteria* (E) from qRT-PCR and RNA Amplicon-seq dataset, and *Bacteroidetes* (C) and *Gammaproteobacteria* (F) from qPCR and DNA Amplicon-seq dataset.

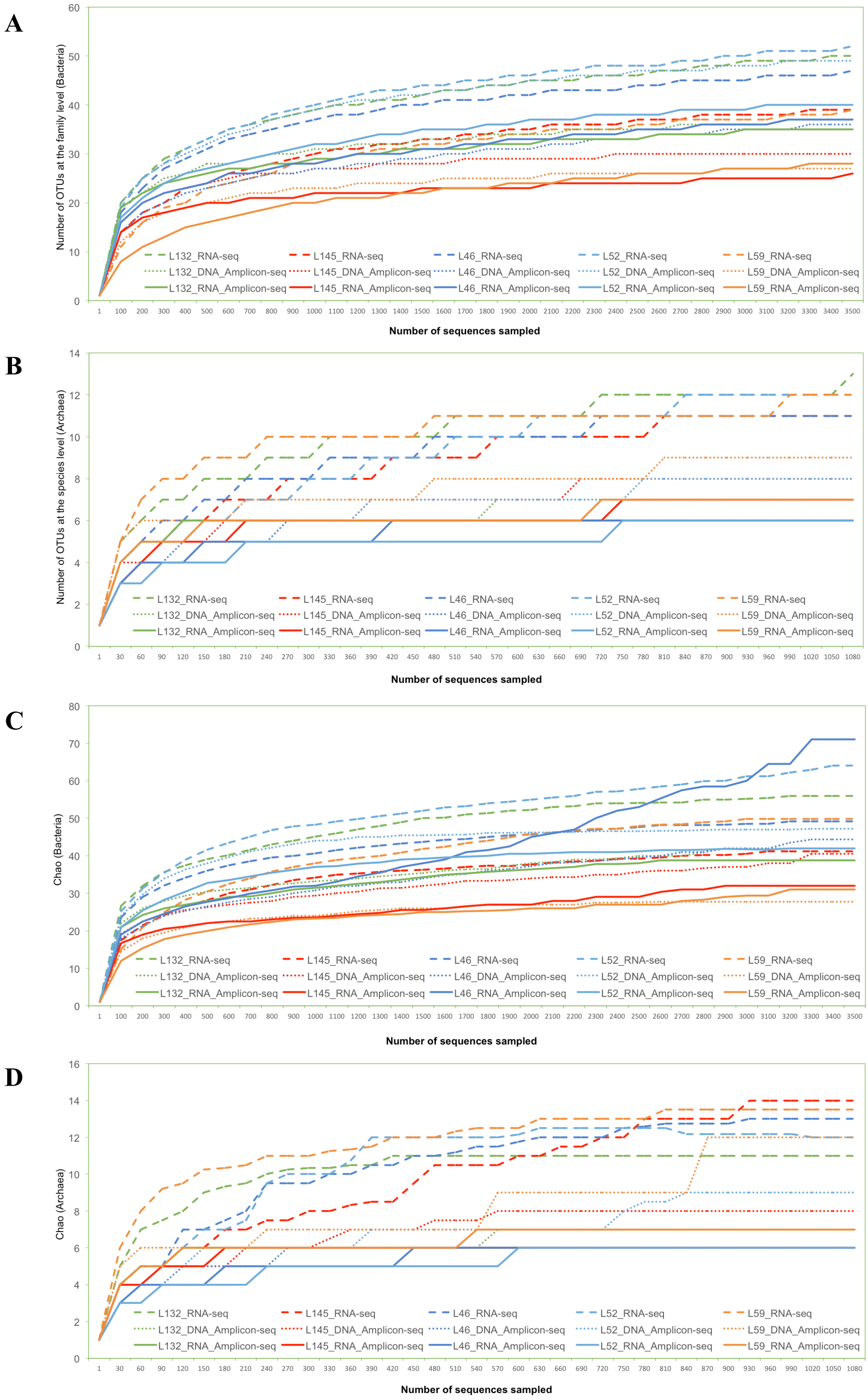

SUPPLEMENTARY FIGURE S2 Rarefaction analysis of rumen bacteria and archaea. Collector's curves for (A) the number of bacterial phylotypes at the family level (B) the number of archaea phylotypes at the species level (C) Chao based on bacterial phylotypes at the family level (D) Chao based on archaeal phylotypes at the species level. The number of reads in each sample was randomly normalized to 3,476 for bacteria and 1,074 for archaea.

References

Muhling, M., Woolven-Allen, J., Murrell, J.C., and Joint, I. (2008). Improved group-specific PCR primers for denaturing gradient gel electrophoresis analysis of the genetic diversity of complex microbial communities. *Isme j* 2**,** 379-392. doi: 10.1038/ismej.2007.97.

Schwieger, F., and Tebbe, C.C. (1998). A new approach to utilize PCR-single-strand-conformation polymorphism for 16S rRNA gene-based microbial community analysis. *Appl Environ Microbiol* 64**,** 4870-4876.

Stevenson, D.M., and Weimer, P.J. (2007). Dominance of Prevotella and low abundance of classical ruminal bacterial species in the bovine rumen revealed by relative quantification real-time PCR. *Appl Microbiol Biotechnol* 75**,** 165-174. doi: 10.1007/s00253-006-0802-y.
